# Neuroinvasive West Nile Encephalitis Presenting With Seizures and Acute Flaccid Paralysis: A Case Report

**DOI:** 10.7759/cureus.80163

**Published:** 2025-03-06

**Authors:** Bola Habeb, Kandace Williams, Nilgun Demirag, Sandy Khair, Seth Fowler

**Affiliations:** 1 Department of Internal Medicine, University of Florida College of Medicine/Ascension Sacred Heart, Pensacola, USA; 2 Department of Radiation Oncology, Cairo University, National Cancer Institute, Cairo, EGY

**Keywords:** long-term neurological outcomes, neuroinvasive west nile virus, west nile-associated acute flaccid paralysis, west nile associated rash, west nile-associated seizures, west nile fever, west nile virus encephalopathy, west nile virus epidemiology

## Abstract

West Nile encephalitis (WNE) is a serious neurological disorder caused by the West Nile virus (WNV), a mosquito-borne flavivirus. The virus is mainly transmitted to humans through bites from infected Culex mosquitoes, with birds acting as the primary reservoir hosts. While most WNV infections are asymptomatic or cause a mild febrile illness (West Nile fever), a small subset of cases progresses to severe neuroinvasive disease, such as encephalitis, meningitis, or acute flaccid paralysis. WNE is marked by brain inflammation, resulting in high fever, headache, neck stiffness, disorientation, tremors, seizures, and paralysis. Advanced age and immunocompromised states are important risk factors for severe disease. There is no specific antiviral treatment for WNE, and management remains supportive. Preventive measures, such as mosquito control and public awareness, are essential for reducing the incidence of this disease. WNV has a global distribution, with outbreaks reported in North America, Europe, Africa, and Asia, making it a significant public health issue.

## Introduction

West Nile virus (WNV) is a mosquito-borne flavivirus that has become a significant cause of viral encephalitis across various regions worldwide. Initially identified in 1937 in the West Nile Province of Uganda, it was first detected in the United States in 1999 [1,2]. While the majority of infections are asymptomatic or result in mild flu-like symptoms, less than 1% of cases, particularly among the elderly and immunocompromised, progress to severe neuroinvasive disease [1]. West Nile encephalopathy (WNE) is one of the most severe neurological manifestations of WNV infection, often presenting with altered mental status, receptive aphasia, movement disorders, and seizures. Although seizures are uncommon in WNV neuroinvasive disease, they have been documented in cases with extensive CNS involvement. Another key feature of severe WNV infection is acute flaccid paralysis, which arises from viral-mediated destruction of anterior horn cells in the spinal cord, resembling poliomyelitis [1]. The simultaneous occurrence of encephalopathy, seizures, and flaccid paralysis is rare but represents a severe clinical presentation associated with significant morbidity.

WNV infection is diagnosed primarily through serologic testing, including detection of IgM antibodies and polymerase chain reaction (PCR) testing, which can confirm the presence of the virus in CSF or blood. Management of WNV infection is largely supportive, as no specific antiviral treatments are available. Supportive care may include IV fluids, analgesics, antipyretics for symptom control, and anticonvulsants for seizure management. In severe cases with neuroinvasive disease, close monitoring of respiratory and cardiac functions, aspiration precautions, and physical and occupational therapy are critical. Prevention involves mosquito control measures, such as the use of insect repellent, protective clothing, and mosquito nets [3].

Here, we present a case of WNE in an adult patient who developed recurrent seizures, expressive aphasia, and profound flaccid paralysis. This report highlights the importance of considering WNV in the differential diagnosis of encephalopathy with neurological deficits, particularly in endemic areas or during peak transmission seasons. Early recognition and supportive management are crucial in improving patient outcomes.

## Case presentation

**Figure 1:**
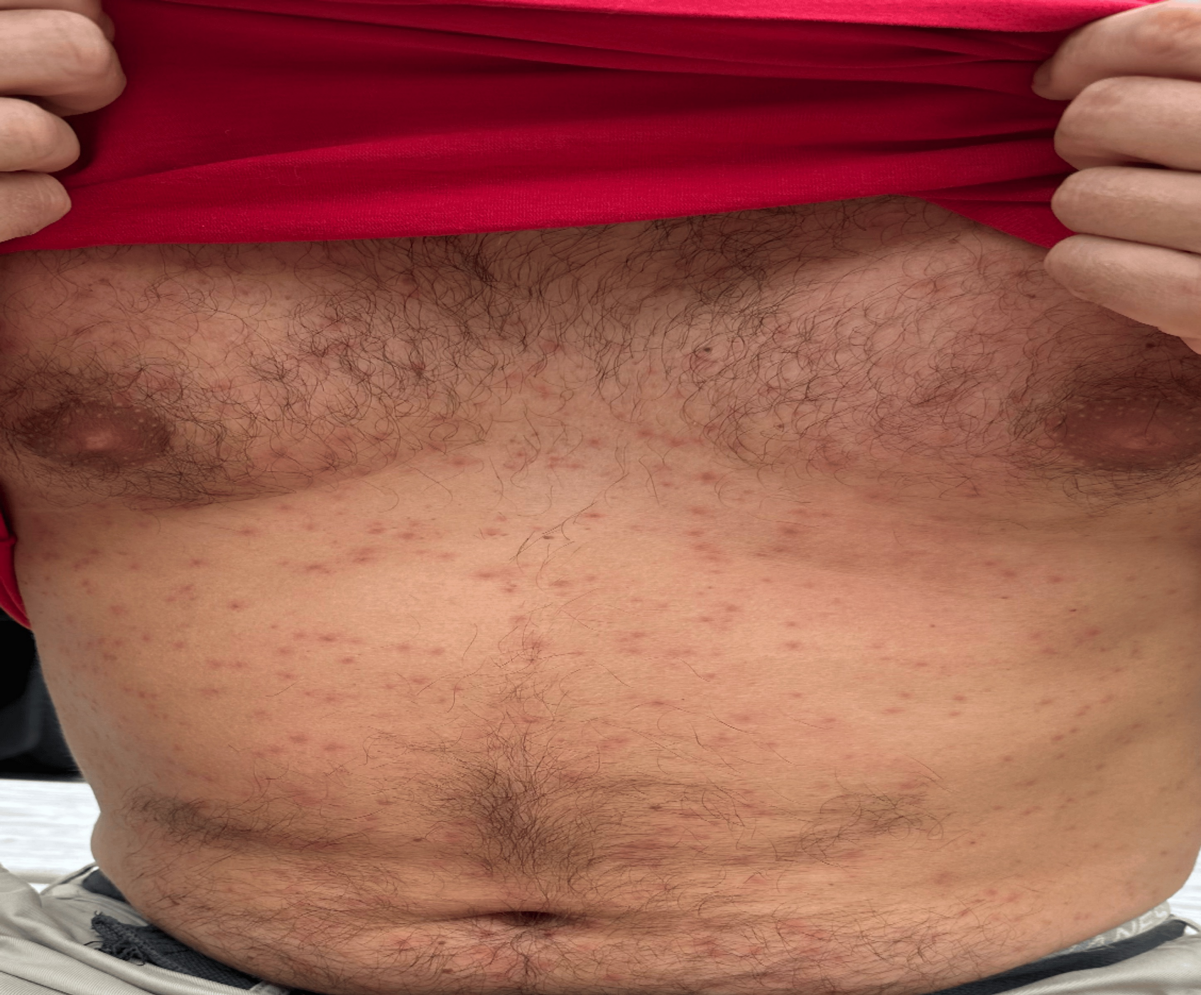
Diffuse maculopapular rash on the chest and abdomen of a patient with West Nile virus.

A 41-year-old male with a medical history of hypertension and anxiety disorder was admitted to our facility for further evaluation following a three-day episode of malaise, low-grade fever, runny nose, and a diffuse maculopapular rash (Figure 1).

The patient resided near a river basin in the southeastern United States, a region with a high mosquito population but not known to be endemic for WNV infection. However, he did not recall any recent mosquito or tick bites. Subsequently, within the first 24 hours, his mental status deteriorated, resulting in confusion and the onset of generalized tonic-clonic seizures.

Vital signs on admission revealed a temperature of 38.7°C, blood pressure of 142/88 mmHg, a pulse of 92 beats per minute (bpm), 18 breaths per minute, and an oxygen saturation of 99% on ambient air. On examination, the patient was awake but drowsy, with fluctuating levels of consciousness despite the correction of his hyponatremia. A thorough neurological exam, including cranial nerve assessment, was unremarkable, with no focal neurological deficits observed. Laboratory data on admission are demonstrated in Table 1.

**Table 1:** Laboratory data obtained on admission. BUN: Blood urea nitrogen; AST: Aspartate aminotransferase; ALT: Alanine aminotransferase; TSH: Thyroid-stimulating hormone; HIV Ag, Ab: Human immunodeficiency virus antigen, antibody; RPR Ql: Rapid plasma reagin qualitative test.

Parameters	Reference Range, Adults	Patient's Values on Admission
Hemoglobin (g/dL)	12.0-15.5	13.7
Hematocrit (%)	34.9-44.5	39.4
White cell count (per mm³)	3,500-10,500	13,500
Platelet count (per mm³)	150,000-450,000	104,000
Sodium (mEq/L)	135-145	127
Potassium (mEq/L)	3.5-5.1	3.4
Bicarbonate (mEq/L)	22-29	21
BUN (mg/dL)	12-21	15
Creatinine (mg/dL)	0.7-1.2	1.33
Ionized calcium (mg/dL)	4.7-5.4	4.6
Lactate (mmol/L)	0.9-1.7	1.3
AST (units/L)	12-31	37
ALT (units/L)	9-29	181
Total bilirubin (mg/dL)	0.1-1.1	0.9
TSH (mcIU/mL)	0.35-4.9	1.15
Ammonia (µmol/L)	18-72	43
HIV Ag, Ab combo screen	Negative	Negative
RPR Ql	Non-reactive	Non-reactive
Lyme disease IgM/IgG	Negative	Negative

An initial workup was performed, which included a CT scan of the brain and computed tomography angiography (CTA) of the head and neck, both of which were unremarkable. The patient also underwent a lumbar puncture and CSF analysis. Empiric therapy for encephalitis or meningitis was initiated with IV vancomycin, ceftriaxone, acyclovir, and dexamethasone. The CSF interpretation revealed an elevated white blood cell count with a predominant lymphocytic composition. The findings included normal glucose levels, elevated protein, and a negative WNV IgM result (Table 2).

**Table 2:** CSF analysis. WNV IgM: West Nile virus immunoglobulin M; WNV IgG: West Nile virus immunoglobulin G.

Parameter	Normal Value Range	Patient’s Value
Color	Colorless	Colorless
Appearance	Clear	Clear
CSF Tube Number	#2	#2
CSF WBC Count	0-6 x 10^6^/L	135 x 10^6^/L
CSF RBC Count	0 x 10^6^/L	161 x 10^6^/L
Segmented Neutrophil CSF	0-6%	29%
Lymphocyte CSF	40-80%	63%
Monocyte/Macrophage CSF	15-45%	8%
CSF Glucose (mg/dL)	40-70	65
CSF Protein (mg/dL)	15-40	73.1
WNV IgM CSF	Negative	Negative
WNV IgG CSF	Negative	Negative

**Figure 2:**
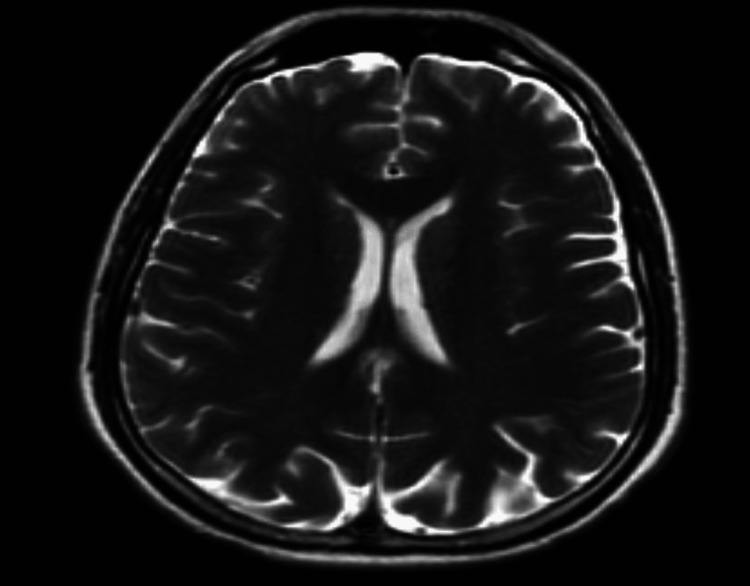
Initial brain MRI of a patient with West Nile encephalitis showing no abnormalities.

Additionally, the patient underwent MRI of the brain, which showed no evidence of intracranial pathology (Figure 2).

**Figure 3:**
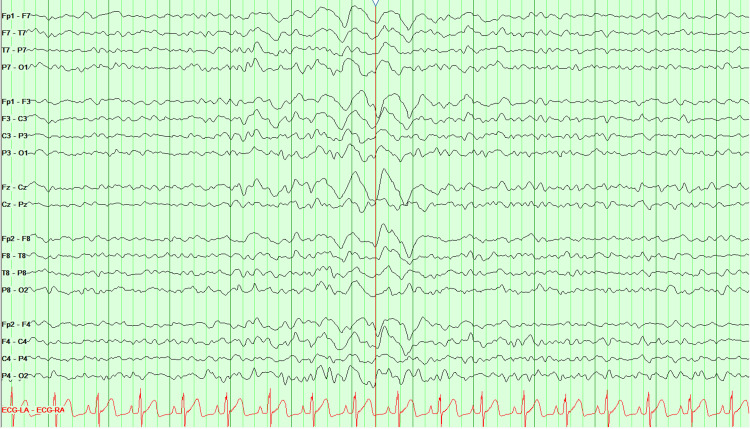
EEG showing intermittent episodes of generalized rhythmic delta activity (GRDA), consistent with metabolic encephalopathy, and without any signs of epileptogenicity.

A continuous video EEG revealed intermittent episodes of generalized rhythmic delta activity (GRDA), consistent with metabolic encephalopathy, without any signs of epileptogenicity or electrographic seizure activity (Figure 3).

Despite negative serologic and imaging results, the patient’s encephalopathy persisted. On day three, he developed acute flaccid paralysis, expressive aphasia, and dysphagia. Given his clinical deterioration and the high suspicion of a mosquito-borne infection, such as WNV, based on his area of residence, a follow-up brain MRI was performed, showing no significant changes. A repeat lumbar puncture was also conducted, revealing similar findings, including an elevated white blood cell count with lymphocytic predominance, normal glucose levels, and elevated protein. Notably, WNV IgM and PCR tests returned positive, confirming the diagnosis of WNE with neuroinvasive disease.

As a result, antibiotics were discontinued, and the patient received supportive care, including acetaminophen for fever and headaches, IV fluids to prevent dehydration, and IV levetiracetam 500 mg twice daily for seizure management. Preventive measures were implemented, including aspiration precautions, venous thromboembolism prophylaxis, and close airway monitoring. The patient also participated in comprehensive physical and occupational therapy, as well as speech and swallowing evaluations. His condition gradually improved, and he was able to ambulate with assistance. He was discharged with instructions to continue antiepileptic medications, refrain from driving for six months until cleared by his neurologist, and pursue ongoing physical and cognitive rehabilitation.

## Discussion

WNV is an arthropod-borne virus (arbovirus) primarily transmitted to humans through the bite of infected Culex mosquitoes. The virus was first identified in the West Nile region of Uganda in 1937 and has since spread globally [1]. In the United States, WNV was first detected in 1999, where it quickly became one of the most common mosquito-borne viral diseases [2]. The virus is endemic in many parts of the world, including Africa, Europe, Asia, and the Middle East. In the United States, most cases occur during the late summer and fall, when mosquito populations are most active. The primary hosts of WNV are birds, with mosquitoes serving as vectors that transmit the virus to humans and other mammals, such as horses. While the virus is most commonly spread via mosquito bites, other transmission routes, such as blood transfusions, organ transplants, and in rare cases, vertical transmission from mother to fetus, have been documented [4].

Risk factors for severe WNV infection include advanced age, immunocompromised status, and underlying health conditions such as diabetes and hypertension. Although most individuals infected with WNV remain asymptomatic, a small subset will develop more severe neuroinvasive disease, particularly in more vulnerable populations, such as the elderly or those with weakened immune systems.

The clinical presentation of WNV infection ranges from mild to severe. Approximately 80% of individuals infected with WNV are asymptomatic, while around 20% will develop mild symptoms, typically characterized by West Nile fever. This illness presents with nonspecific symptoms, including fever, headache, muscle aches, fatigue, rash, and swollen lymph nodes. Symptoms usually resolve within a few days to a week, and patients generally recover completely [5]. In approximately 1 in 150 infected individuals, WNV leads to severe neuroinvasive disease, which can manifest as encephalitis (brain inflammation), meningitis (inflammation of the membranes surrounding the brain and spinal cord), or meningoencephalitis (a combination of both) [4]. The most concerning complication is acute flaccid paralysis, which may present with sudden onset muscle weakness or paralysis, often in an asymmetric pattern. This paralysis is typically seen in the limbs, and in severe cases, it can affect respiratory muscles, leading to respiratory failure and requiring mechanical ventilation. Patients with neuroinvasive disease often experience neurological symptoms such as seizures, confusion, altered mental status, and focal neurological deficits.

The diagnosis of WNV infection is based on clinical suspicion, patient history, and laboratory testing. For patients presenting with neurological symptoms, laboratory tests are crucial to confirm the diagnosis. Serological testing for WNV IgM antibodies in blood or CSF is the primary diagnostic tool, with the presence of IgM antibodies in CSF being highly indicative of neuroinvasive WNV infection. Typically, CSF findings reveal a mildly elevated WBC count (pleocytosis), predominantly lymphocytes, along with an elevated protein concentration. Glucose levels in the CSF are usually normal. Initial serological testing for WNV infection may yield false-negative results, particularly in immunocompromised patients. If clinical suspicion for WNV infection remains high despite negative initial serology within the first 7-10 days of symptom onset, repeat CSF testing may be warranted [6]. PCR testing can also be used to detect viral RNA in blood or CSF, particularly in the early stages of infection. Neuroimaging, such as MRI, may show nonspecific changes, including cortical or subcortical lesions, but imaging is not typically diagnostic in almost 30% of cases [6]. Electromyography (EMG) and nerve conduction studies may be performed in patients with acute flaccid paralysis to assess the extent of motor nerve involvement and help differentiate WNV-induced paralysis from other conditions, such as Guillain-Barré syndrome.

Management of WNV primarily involves supportive care, as there is no specific antiviral therapy available [7]. For patients with mild West Nile fever, outpatient treatment is often sufficient. Symptomatic treatment includes rest, hydration, and the use of antipyretics like acetaminophen to control fever and pain. Most patients recover completely without the need for hospitalization [7]. On the other hand, for patients with severe neuroinvasive disease, hospitalization is typically required. Supportive care for neuroinvasive WNV includes management of fever, seizure control, and pain relief, as well as close monitoring of neurological status. Seizures may be managed with anticonvulsants, and respiratory support, including mechanical ventilation, may be necessary for those with severe respiratory muscle weakness. Patients with acute flaccid paralysis may benefit from physical therapy to maintain joint mobility and reduce the risk of contractures. In addition, rehabilitation through physical, occupational, and speech therapy may be essential for patients with long-term neurological deficits, particularly those who suffer significant motor weakness or cognitive impairments.

The prognosis for WNV infection depends on the severity of the illness and the presence of neuroinvasive disease. Most individuals with mild West Nile fever recover completely within a few weeks, and the prognosis is excellent. However, for those who develop neuroinvasive disease, the outcome is more variable. A study assessing patients an average of 13 months post-infection found that 49% reported fatigue, 24% experienced depression, 20% had new tremors, and 24% faced moderate-to-severe disability [8]. Mortality rates for severe neuroinvasive disease range from 10-20%, and the likelihood of severe outcomes is higher in older adults and immunocompromised individuals [7]. The severity of symptoms at presentation, such as coma or the need for mechanical ventilation, is a strong predictor of poor prognosis [7].

Among survivors of neuroinvasive WNV infection, long-term sequelae may include persistent neurological deficits. While some individuals with acute flaccid paralysis experience gradual recovery over months to years, others may have lasting weakness or permanent disability. Cognitive impairments, such as memory deficits and difficulty with concentration, can persist, and many patients face psychological challenges, including depression and anxiety. Comprehensive rehabilitation, including physical and cognitive therapy, plays a critical role in improving functional outcomes; however, the recovery process is often prolonged, and some individuals may require long-term supportive care [9].

Consequently, early identification of WNV infection is essential for optimizing patient outcomes, particularly in those at risk for severe complications such as encephalitis, meningitis, or acute flaccid paralysis. Timely diagnosis allows for close monitoring and early interventions, including respiratory support and seizure management, while also preventing unnecessary use of empiric antibiotics or antivirals for alternative causes of encephalopathy. From a public health standpoint, early detection facilitates outbreak surveillance and mosquito control efforts, helping to mitigate further transmission. Furthermore, an accurate diagnosis informs prognosis and long-term care planning, as patients with neuroinvasive disease often require rehabilitation and supportive therapy for extended recovery.

## Conclusions

Encephalopathy, seizures, and acute flaccid paralysis are severe and complicated forms of WNV infection. While the majority of individuals infected with the virus are either asymptomatic or experience mild, flu-like symptoms, those who progress to neuroinvasive disease can face serious neurological issues such as seizures, confusion, altered mental status, and focal neurological deficits. The onset of seizures and acute flaccid paralysis is especially concerning, as these complications can lead to long-lasting disability, respiratory failure, and even death. Early diagnosis of WNV infection is crucial despite the absence of specific antiviral treatment because it allows for appropriate supportive care, prevents unnecessary interventions, and aids in public health efforts. Management primarily involves symptom control, preventing further complications, and addressing the patient's rehabilitation needs.

The prognosis for individuals with neuroinvasive WNE is variable, with many patients experiencing significant long-term neurological deficits such as weakness, cognitive dysfunction, and emotional challenges. Mortality rates for severe cases are elevated, particularly among older adults and those with compromised immune systems. However, some patients can recover with proper rehabilitation, which helps in improving motor function and overall quality of life. While recovery can be slow, early intervention and rehabilitation therapies are key factors in optimizing functional outcomes. Furthermore, ongoing research and prevention measures, such as mosquito control, are vital in mitigating the impact of WNV infections.
